# Optimizing Ankle Sprain Management in Primary Care: A Randomized Trial of Telerehabilitation Added to Usual Care

**DOI:** 10.7759/cureus.86664

**Published:** 2025-06-24

**Authors:** Juan Figueroa-García, Víctor Granados-García, Silvia Martínez-Valverde, Guillermo Salinas-Escudero, Juan Carlos H Hernández-Rivera, David Rojano-Mejía

**Affiliations:** 1 Centro de Investigación Educativa y Formación Docente, OOAD Sur de la Ciudad de México, Instituto Mexicano del Seguro Social, Mexico City, MEX; 2 Unidad de investigación epidemiológica y en servicios de salud, área envejecimiento, Instituto Mexicano del Seguro Social, Mexico City, MEX; 3 Centro de Estudios Económicos y Sociales en Salud, Hospital infantil de México Federico Gómez, Instituto Nacional de Salud, Mexico City, MEX; 4 Centro de Estudios Económicos y Sociales en Salud, Hospital Infantil de México, Instituto Nacional de Salud, Mexico City, MEX; 5 Unidad de Investigación Médica en Enfermedades Nefrológicas, Hospital de Especialidades, Centro Médico Nacional Siglo XXI, Instituto Mexicano del Seguro Social, Mexico City, MEX; 6 Coordinación de Investigación en Salud, Instituto Mexicano del Seguro Social, Mexico City, MEX

**Keywords:** ankle sprain, effectiveness, musculoskeletal diseases, telemedicine, telerehabilitation

## Abstract

Background: Ankle sprains (AS) are among the most common musculoskeletal injuries, with physical rehabilitation being a cornerstone of treatment. Telerehabilitation has emerged as an effective alternative for managing various musculoskeletal conditions; however, evidence supporting its use specifically for AS remains limited. This study aimed to evaluate whether the addition of structured telerehabilitation to usual care (UC) improves functional recovery in patients with grade I-II AS more effectively than UC alone in a primary care setting.

Methods: Eighty-two participants were randomized into two groups (41 each): 1) Intervention group (IG): UC (standard primary care management) plus a four-week telerehabilitation program (30-minute daily exercises, five days/week) delivered via a digital platform with pre-recorded videos; Control group (CG): UC only. The primary outcome was ankle functionality measured using the Foot and Ankle Ability Measure (FAAM), including subscales for activities of daily living (FAAM-ADL) and sports (FAAM-Sports). Secondary outcomes included pain perception and work disability days.

Results: At four weeks, the IG demonstrated superior outcomes; at functionality, mean between-group differences favored the IG (FAAM-ADL: +8.3 points [95% CI 3.8-12.7]; FAAM-Sports: +13.4 points (6.7-20)). Pain: Significant reduction in VAS scores for grade II AS only (−0.9 points (−1.5 to −0.4)). Subgroup analysis: Clinically meaningful improvements in functionality were observed for both grades, but pain reduction was significant only in grade II AS. Grade I AS showed improvement limited to FAAM-Sports.

Conclusions: Telerehabilitation, as an adjunct to UC, significantly enhances functional recovery in AS, with particularly pronounced effects in grade II (moderate) sprains. However, pain reduction exhibits grade-dependent variability. These findings support incorporating telerehabilitation into primary care management protocols for AS.

## Introduction

Ankle sprains (AS) are among the most prevalent musculoskeletal injuries [1], with significant variability in reported incidence across different populations [2,3]. They represent a leading cause of physical disability and work absenteeism, yet only 50% of affected individuals seek medical care [4]. Without proper treatment, AS can lead to chronic complications and permanent functional impairment [1,4]. Current management strategies often fail to provide optimal rehabilitation for all patients [1], despite their critical role in recovery.

Rehabilitation focusing on strength, range of motion, and proprioception is essential for functional recovery and reducing recurrence [4,5]. Standard care typically includes RICE protocol (rest, ice, compression, elevation) and NSAIDs [6]. However, only 6.8% of AS patients complete prescribed rehabilitation programs [7], highlighting a critical gap in care delivery.

Telerehabilitation has emerged as an effective solution for various musculoskeletal conditions [8], but its specific benefits for AS remain understudied [9]. This randomized trial evaluated whether adding structured telerehabilitation to usual care (UC) improves functional outcomes in grade I-II AS, compared to UC alone in primary care settings. We hypothesized that the combined approach would demonstrate superior effectiveness, particularly in functional recovery measures.

## Materials and methods

Design

We conducted a pragmatic, evaluator- and analyst-blinded, randomized clinical trial with two parallel arms, following the CONSORT guidelines.

This randomized clinical trial was conducted in accordance with the ethical principles established by the Declaration of Helsinki and followed national and international ethical guidelines for research involving human participants. The study protocol was reviewed and approved by the local ethics and health research committee of the institution where the study was conducted (registry and approval number: R-2021-3609-037), and registered at clinicaltrials.gov (identifier: ID- NCT05217173). All participants provided written informed consent after receiving detailed information about the study’s objectives, procedures, and potential risks and benefits. Confidentiality and data protection were ensured in compliance with applicable regulations.

Participants were randomly allocated (1:1) to the intervention group (IG) or control group (CG) using a computer-generated randomization list created in Excel (Microsoft for MacOS). Numbers 1 (IG) and 2 (CG) were each repeated 41 times in a column. A second column with randomly generated numbers was sorted in descending order to determine the allocation sequence. The randomization list was kept confidential and controlled by the principal investigator. Group assignment was communicated verbally to collaborators as each participant enrolled in the study.

Physicians providing UC were blinded to the group assignment of the participants. However, since participants needed to know the instructions for their respective treatment groups, and the evaluators needed to provide these instructions, it was impossible to blind both parties initially. In the subsequent evaluations, different evaluators, who were unaware of the participants’ treatment groups, conducted the assessments, this action was implemented to mitigate potential bias in outcome measurement and to prevent the recording of results from being influenced by the evaluator's knowledge of the study group to which the subject belonged. The data analyst remained blinded throughout the study.

Participants

Eligible participants were adults (18-60 years) diagnosed with grade I or II AS within 72 hours by a family medicine specialist. Imaging studies were not required, as per primary care standards in our setting. Potential participants were identified through daily diagnostic logs from the primary care facility, contacted via telephone, and screened for eligibility; if they were willing to volunteer to participate in the study, a personal interview was scheduled for the next day; a research collaborator evaluated the inclusion criteria. Those who met the selection criteria were asked to sign the informed consent form to participate in the study. The inclusion criteria were as follows: individuals diagnosed with grade I or II AS (as grade III AS are not treated in primary care units in our environment), of any anatomical location, aged between 18 and 60 years, capable of using a mobile phone or computer with internet access, and within 72 hours of sustaining the sprain. The exclusion criteria included individuals with a history of previous AS, central or peripheral neurological disorders, leg or foot ulcers, fibromyalgia, pre-existing ankle arthritis, significant ipsilateral or contralateral lower extremity injury, and those using corticosteroid medications (oral or intravenous).

Intervention and control groups

The CG consisted of people receiving UC. UC consisted of clinical evaluation of the diagnosis and pharmacological treatment, which did not include rehabilitation. The IG received UC and telerehabilitation. The IG participants accessed the telerehabilitation content via mobile phone and/or computer through an application (MoodleCloudTM). The content of the application consisted of basic information on AS and previously recorded video rehabilitation exercises. They were instructed to perform the rehabilitation exercises featured in the videos for 30 minutes/day, five days/week for four weeks. This time is what is considered adequate to return to daily activities after an AS [10]. It was also explained to them that the telerehabilitation exercises consisted of three modules (Appendix 1). The rehabilitation exercises of weeks one and two focused on proprioception and movement, and those of weeks three and four focused mainly on strength exercises.

Outcome measures

Primary Outcome

All data were recorded face-to-face in the clinic using pencil and paper. The primary measurements were performed five times, at the beginning of the study (baseline) and once each subsequent week. Ankle functionality in both groups was measured using the Foot and Ankle Ability Measure (FAAM) instrument. The FAAM at week four was considered the primary outcome measure for assessing ankle functionality, providing a validated and reliable indicator of patient progress. The FAAM consists of two subscales: Activities of Daily Living (FAAM-ADL) with 21 items, and Sports Activities (FAAM-SA) with 8 items. Each item is scored from 0 (unable to perform the activity) to 4 (no difficulty at all). The final score is transformed into percentage scores from 0 (lower functionality) to 100 (higher level of functionality) [11].

Secondary Outcome

Pain perception was measured through the visual analog scale (VAS-pain). For the VAS-pain, a scale of 0 to 10 (divided in cm) was used, where 0 is the absence of pain and 10 is the most intense pain that the person can experience. We also asked about the use of other therapeutic actions at any time in the four weeks (use of ice, elevation of the ankle, use of NSAIDs). Another measure was the quantification of days of work incapacity (representing the number of days it takes a person to return to work after suffering a sprain).

Data analysis

The results of a study that measured the effect of rehabilitation on ankle functionality (through the FAAM) were taken as a reference; the difference obtained between before and after rehabilitation was 9.1 in the FAAM-ADL subscale, with a standard deviation of 11.07 [12]. The values for the calculation were 90% power, 20% loss, and <0.05 for alpha, with a sample of 41 participants per group. The data were analyzed using the intention-to-treat principle. Missing data from participants without follow-up were imputed by predictive mean matching. The Shapiro-Wilk test was performed to determine the normality of data distribution. When the assumption of normality was not met, the median and interquartile ranges were used. A comparison of the dependent variable results was conducted using the Mann-Whitney U test, while weekly within-group data were analyzed with the Friedman test. The associations of the variables with the distribution of the results by groups were evaluated with a chi-square test, and p <0.05 was considered to indicate statistical significance. All analyses were performed using SPSS (Statistical Package for the Social Sciences, version 29, IBM Corp, Armonk, NY, United States). Linear mixed models (LMM) were used to compare changes in FAAM-ADL, FAAM-Sports, and VAS-pain scores over the four-week follow-up. The models included random effects of participants and fixed effects of group, time, and interaction between group and time.

## Results

The team recruited participants between February and October 2022. The study sample consisted of 82 participants, 41 in the IG and 41 in the CG. The mean age in both groups was similar (35.2 for IG vs 36.8 for CG; p 0.3). The IG had a greater proportion of people with a higher educational level. There were more overweight participants in the IG and obese participants in the CG. More than half of the participants had a diagnosis of grade 2 of AS (61%) (Table 1).

At the end of the study, both groups showed improvements in ankle functionality and decreased pain; However, the IG achieved greater improvements in functionality with FAAM-ADL scores of 71 points vs 62.2 points in the CG, in FAAM-sports activities (SA) scores of 71.7 vs 57.4 and VAS-pain scores with a decrease in pain of 7.3 vs 6.5 points, respectively (Table 2).

**Table 1:** Demographic and clinical characteristics of the sample SD: standard deviation; BMI: body mass index For qualitative variables, the chi-square test was employed.

Measure	Intervention Group (n=41)	Control Group (n=41)	p-value
Age group, frequency (%)			
18-30	18 (43.9%)	14 (34.1%)	0.365
31-40	12 (29.3%)	10 (24.4%)	0.618
41-50	6 (14.6%)	10 (24.4%)	0.265
51-60	5 (12.2%)	7 (17.1%)	0.532
Sex, frequency (%)			
Female	18 (43.9%)	22 (53.7%)	0.377
Male	23 (56.1%)	19 (46.3%)	0.377
Education, frequency (%)			
Primary and secondary school	8 (19.5%)	17 (41.5%)	0.031
High school	17 (41.5%)	18 (43.9%)	0.823
Higher education	16 (39%)	6 (14.6%)	0.013
BMI classification, frequency (%)			
Underweight	4 (9.8%)	2 (4.9%)	0.396
Normal	14 (34.1%)	15 (36.6%)	0.817
Overweight	13 (31.7%)	8 (19.5%)	0.206
Obesity	10 (24.4%)	16 (39%)	0.154
Ankle sprain grade, frequency (%)			
Grade I	20 (48.8%)	12 (29.3%)	0.07
Grade II	21 (51.2%)	29 (70.7%)	0.7

**Table 2:** Mean (95% CI) for outcomes of FAAM-ADL, FAAM-SA, and VAS-pain for each group, mean (95% CI) difference within groups, and mean (95% CI) difference between groups IG: intervention group; CG: control group; FAAM: foot and ankle ability measure; ADL: activities of daily living; SA: sports activities; VAS-pain, visual analog scale for pain.

Study groups and assessment points										
Outcome	Basal		Week 1		Week 2		Week 3		Week 4	
	IG (n=42)	CG (n=42)	IG (n=42)	CG (n=42)	IG (n=42)	CG (n=42)	IG (n=42)	CG (n=42)	IG (n=42)	CG (n=42)
FAAM-ADL	18.7 (13.9 to 23.6)	18.8 (13.9 to 23.7)	39.8 (34.4 to 45.2)	32.5 (27.1 to 37.9)	58.7 (53.7 to 64)	50.6 (45.4 to 55.7)	75.9 (71.5 to 80.3)	67.6 (63.2 to 72)	89.8 (86.7 to 92.9)	81.5(78.3 to 84.6)
FAAM-SA	6.3 (3.5 to 9.1)	7.1 (4.3 to 9.9)	24.2 (19.9 to 28.4)	18.8 (14.5 to 23.1)	35.8 (31.1 to 40.6)	29.7 (24.9 to 34.3)	56.8 (51.7 to 62)	47.7 (42.5 to 52.8)	78 (73.3 to 82.7)	64.6 (59.9 to 69.3)
VAS-pain	8.4 (8.1 to 8.8)	8.6 (8.2 to 8.9)	6.4 (5.9 to 6.9)	7 (6.5 to 7.5)	4.7 (4.2 to 5.2)	5.6 (5 to 6.1)	2.8 (2.4 to 3.3)	3.8 (3.3 to 4.2)	1.1 (0.7 to 1.5)	2.1 (1.7 to 2.5)
Within-groups difference										
Outcome	1st week minus basal		2nd week minus basal		3rd week minus basal		4th week minus basal			
	IG	CG	IG	CG	IG	CG	IG	CG		
FAAM-ADL	21 (16 to 26) p <0.001	13.7 (8.7 to 18.7) p <0.001	40 (34.4 to 45.7) p <0.001	31.7 (26.1 to37.3) p <0.001	57.1 (51.8 to 62.5) p <0.001	48.7 (43.4 to 54.1) p <0.001	71 (66.1 to 75.9) p <0.001	62.2 (57.7 to 67.5) p <0.001		
FAAM-SA	17.8 (14.5 to 21.0) p <0.001	11.6 (8.4 to 14.9) p <0.001	29.5 (25.5 to 33.4) p <0.001	22.5 (18.6 to 26.4) p <0.001	50.2 (45.7 to 55.3) p <0.001	40.5 (35.7 to 45.3) p <0.001	71.7 (66.9 to 76.4) p <0.001	57.4 (52.6 to 62.2) p <0.001		
VAS-pain	-2 (-2.3 to -1.7) p <0.001	-1.6 (-1.9 to -1.3) p <0.001	-3.7 (-4.1 to -3.3) p <0.001	-3 (-3.4 to -2.6) p <0.001	-5.6 (-6 to -5.1) p <0.001	-4.8 (-5.2 to -4.3) p <0.001	-7.3 (-7.7 to -6.9) p <0.001	-6.5 (-6.9 to -6) p <0.001		
Between-group difference										
Outcome	1st week minus basal		2nd week minus basal		3rd week minus basal		4th week minus basal			
	IG minus CG		IG minus CG		IG minus CG		IG minus CG			
FAAM-ADL	7.3 (-0.3 to 14.9) p=0.06		8.2 (0.9 to 15.5) p=0.02		8.3 (2.1 to 14.5) p=0.009		8.3 (3.8 to 12.7) p <0.001			
FAAM-SA	5.3 (-0.6 to 11.3) p=0.08		6.1 (-0.5 to 12.8) p=0.07		9.1 (1.8 to 16.5) p=0.01		13.4 (6.7 to 20.0) p <0.001			
VAS-pain	-0.5 (-1.2 to 0.1) p=0.1		-0.8 (-1.6 to -0.1) p=0.019		-0.9 (-1.6 to -0.2) p=0.005		-0.9 (-1.5 to -0.4) p <0.001			

The IG had greater use of ice and elevation of the affected ankle; both groups reported the use of NSAIDs and rest similarly (Table 3).

**Table 3:** Therapeutic actions reported by patients at any time during the 4 weeks of follow-up NSAID: non-steroidal anti-inflammatory drugs. The chi-square test was used to determine the independence of the variables.

Measure	Intervention Group (n=41)	Control Group (n=41)	p-value
NSAID use, frequency (%)	39 (95.1%)	41 (100%)	0.247
Ice use, frequency (%)	23 (56.1%)	14 (34.1%)	0.038
Rest, frequency (%)	40 (97.6%)	39 (95.1%)	0.62
Elevation, frequency (%)	23 (56.1%)	11 (26.8%)	0.007

At the conclusion of the study, significant differences favoring the IG were observed in persons with grade 1 AS for the FAAM-Sports subscale, pain measured by VAS, and days of incapacity. However, no notable differences were found between the IG and CG for the FAAM-ADL subscale. In patients with grade II AS, the IG demonstrated significantly greater functional improvement compared to the CG in both FAAM subscales (ADL and Sports), along with lower pain perception measured by VAS and fewer days of incapacity. When the results for both grades of AS were analyzed, the IG consistently outperformed the CG across all measured outcomes, including greater functional recovery, reduced pain perception, and fewer days of incapacity. The Mann-Whitney U test was performed to compare outcomes between the intervention and control groups; statistical significance was observed only at the 4th week for the FAAM-ADL, FAAM-Sports, and days of incapacity in the subgroup of grade II AS, as well as in the combined analysis of both grades. Self-reported telerehabilitation adherence was higher in grade II ankle sprains than in grade I sprains (median: 425 vs 375 minutes) (Table 4).

**Table 4:** Differences in ankle functionality by FAAM, VAS-pain, days of incapacity for work according to the degree of ankle sprain and duration of telerehabilitation exercises FAAM: foot and ankle ability measure; ADL: activities of daily living; VAS-pain: visual analog scale for pain. *p-value <0.05 with the Mann-Whitney U test between the control and intervention groups.

Measure	Intervention Group		Control Group		
Ankle sprain, grade 1	Median (n=20)	IQR	Median (n=12)	IQR	p-value
FAAM score ADL					
Baseline	26	12, 40	21.4	13, 41.1	0.969
Week 4	89.8	81.2, 95.6	85.7	82.7, 95.3	0.572
FAAM score sports					
Baseline	7.75	0, 12.5	7.7	1.5, 18.3	0.753
Week 4	82.7	66.4, 93	70	61.6-78.8	0.119
VAS for pain					
VAS-pain, basal	8	8, 9	8	7.25, 9	0.641
VAS-pain, week 4	1	0, 2	1	1, 2	0.186
Days of incapacity for work	5	4, 7	7	5, 9.7	0.123
Minutes of telerehabilitation performed	375	241, 432	-	-	-
Ankle sprain grade 2	Median (n=21)	IQR	Median (n=29)	IQR	p-value
FAAM score ADL					
Baseline	9.6	4.7, 16.5	13	5.3, 20.6	0.731
Week 4*	93.2	90.2, 95.9	80.9	76.2, 86.3	<0.001
FAAM score sports					
Baseline	0	0, 7.8	3.1	0, 7.1	0.289
Week 4*	81.2	70.1, 85.5	64.2	56.6, 73.5	<0.001
VAS for pain					
VAS-pain, basal	9	8, 10	9	8, 10	0.56
VAS-pain, week 4	1	0, 2	2	1, 3	0.003
Incapacity days for work*	10	7, 14	14	12, 17.5	0.021
Minutes of telerehabilitation performed	425	350, 577	-	-	-

Patient follow-up in the clinical trial

Among the 82 participants recruited, eight from the IG did not complete the follow-up: five due to personal decisions and lack of time for rehabilitation activities, and one due to missing follow-up appointments for functionality evaluation. In the CG, two participants did not complete the activities because they did not respond to phone calls for subsequent evaluation appointments.

## Discussion

Our study investigated the effectiveness of telerehabilitation combined with UC for AS compared to UC alone. By the end of the study, participants in the IG showed significant clinical improvements compared to those in the CG. These improvements included: 1) greater gains in perceived ankle functionality, 2) fewer days of work incapacity, and 3) lower pain perception. These benefits were particularly notable in participants with grade II sprains.

Ankle functionality

Both the IG and CG experienced clinically meaningful improvements in perceived functioning over the four weeks; however, the IG consistently had higher scores than the CG. Despite similar baseline FAAM-ADL scores in both groups, participants receiving telerehabilitation demonstrated greater ankle function throughout all follow-up weeks, with the most notable improvements observed in the first week. Early rehabilitation’s positive impact on ankle functionality has been documented in previous studies [5, 13, 14]. However, our study is unique in comparing telerehabilitation to UC for AS, as no previous reports have been identified.

Despite the improvement in functionality, at the end of the study, no subscale reached 100%. This is important because perhaps participants with AS should have a longer-term follow-up and assess whether they need further medical attention. However, we must also consider that we did not know the ankle functionality of the participants before they sustained the sprain. Our results agree with a study that also performed telerehabilitation for AS, in which clinically significant and favorable results of functional recovery were obtained, although this study differs from ours because this was a cohort study without a control group [9]. It is important to note that there were both similarities and differences between the intervention in the cited study and ours. The similarity was the planned duration of the program, which was designed for four weeks in both cases. However, the differences were notable. In the cited study’s program, the rehabilitation exercises focused solely on improving movement and proprioception, neglecting strength development. The exercises were repetitive, lacked variety, and were not sequential, meaning they did not progress from lower to higher intensity. In contrast, our program incorporated strength development and followed a progressive approach. Another difference was the session duration: in the cited study, sessions lasted 20 minutes daily in the first week, 28 minutes in the second and third weeks, and 29 minutes in the fourth week. In our program, sessions were consistently 30 minutes daily throughout all four weeks. According to patient self-reports, those with grade I sprains completed only 62.5% of the prescribed telerehabilitation duration (median: 375/600 minutes), while those with grade II sprains completed 70.8% (median: 425/600 minutes). This difference in adherence may explain why grade II sprains showed better outcomes compared to the CG.

When analyzing ankle functionality by sprain grade, only minimal clinically important differences were obtained in AS grade II (FAAM minimal clinically important differences are 8 and 9 points for the ADL and Sports subscales, respectively). Therefore, we can say that in grade I sprains, telerehabilitation was not more effective than UC, but in grade II sprains, it was more effective. However, this conclusion is based on the results of our specific trial setting, study methods, and specific interventions.

Days of incapacity for work

The IG had fewer days of work incapacity compared to the CG, resulting in a faster return to work among the IG participants. This finding is clinically significant, as physical disability is one of the main consequences of AS. However, few studies have explored the relationship between telerehabilitation and reduced work incapacity [9, 15]. Existing evidence comes from conventional (face-to-face) rehabilitation studies, which suggest that early initiation of rehabilitation is associated with a quicker return to work or sports activities [14, 16, 17].

There are scientific studies that refer to the number of days of physical disability caused by an AS, but the data reported are not consistent [10, 17, 18]. There is no consensus on the number of days of incapacity to work that should be granted; for instance, some studies indicate that athletes return to sports activities in fewer than 10 days [19], while a systematic review reports a range of 12 to 43 days of work incapacity [20]. Other studies suggest an average of 15 days of work incapacity [21]. This variability in the results could be explained by the different approaches to rehabilitation in each of the investigations. The days of incapacity for work caused by AS are important since they generate economic losses for the days that the affected person is no longer productive [1, 10, 18]. A medical intervention that accelerates return-to-work benefits not only the individual affected by the sprain but also their employer and the economy as a whole.

Perception of pain

In AS, pain interferes with functionality and limits activities of daily living, including work [22]. Pain perception has been used as a parameter to partially evaluate the clinical evolution of AS [5, 21]. One study reported that during the first two weeks after an AS, there is a rapid decrease in pain, after which it continues to improve more slowly [23]. Our results showed a decrease in pain perception in both groups at the four-week follow-up. Although the IG had less pain, this only occurred in patients with grade II sprain, with a difference of one point less than the CG in terms of the VAS-pain score; this difference is considered clinically significant [24]. In addition, only time (weeks) and intervention (only for grade II sprain and when for both grades together) had statistical significance for pain reduction. Some studies have reported that using ice alone does not modify the progression of AS or reduce pain. Significant improvement is only achieved when ice is combined with rehabilitation exercises [4]. However, our results appear to be consistent with those of other clinical trials on pain perception when comparing groups of participants undergoing rehabilitation vs. those receiving UC, as there are changes, but not clinically significant [12, 14]. To be considered clinically significant or to have a minimal clinically important difference, this result must be equal to or greater than 1.3 points on the VAS scale for pain [25]. To clarify, we employed a modified VAS for pain assessment, featuring incremental centimeter markings along the traditional 10-cm line. Although this adaptation maintains the scale's measurement validity, we recognize the importance of explicitly reporting this methodological detail.

Another variable that can be considered essential to assess the evolution of pain was the use of NSAIDs; however, in our study, it is difficult to evaluate this variable, since both groups used them in a similar percentage. Although it is known that NSAIDs decrease pain and are only helpful for a short period, there are no differences in pain perception regardless of the type of NSAID used [6, 26, 27].

Limitations and strengths

The main limitation of this study pertains to the selection criteria for the severity grades and anatomical locations of AS. Despite randomization, homogeneous groups were not obtained, and the CG had a greater proportion of grade II sprains, which may have influenced the gain in functionality and days of incapacity to work for this group. A second limitation was the uncertainty regarding program compliance among IG participants, partly due to the nature of the study (characteristics of a pragmatic clinical trial); measuring compliance was not an objective of this study. It is important to mention that there is no consensus on the number and duration of rehabilitation sessions [28]. However, successful results have been reported for rehabilitation interventions, ranging from 3.5-21 hours, with session durations of 10 to 60 minutes, spread over 5 to 84 sessions [28]. In a study involving telerehabilitation for AS, participants achieved positive outcomes with an average of 750 minutes of therapy over five weeks [9]. Therefore, it would be interesting to conduct future research comparing our telerehabilitation program with other populations. A third limitation was the lack of investigation into the specific work activities of participants, which could have influenced their return-to-work timelines. Finally, our study is relatively innovative and does not allow us to have enough parameters for the comparison of results.

Despite these limitations, our research has several strengths. First, it adhered to CONSORT guidelines, ensuring transparency in the communication of methodology and results [29]. Ankle functionality was assessed using the FAAM, which is the instrument recommended by the 2019 International Ankle Consortium Executive Committee expert panel for the assessment and follow-up of AS [1]. There were no ethical controversies regarding the therapy assigned to the patients since both groups were treated and followed for the duration of the study. Another strong point was that the calculated statistical power was preserved (90%), since the sample loss was less than 20%. Another strength was the study design, which included blinding of evaluators and the data analyst to reduce bias. Finally, this is a relatively innovative study that integrates rehabilitation through telerehabilitation. To our knowledge, no similar studies have been conducted in our setting, making these findings particularly valuable for advancing the field.

## Conclusions

Adding telerehabilitation to treatment for AS improves functional recovery, although this is greater for grade II sprains than for grade 1 sprains. The addition of telerehabilitation for AS results in fewer days of incapacity for work and reduces pain perception compared to UC. Telerehabilitation is a therapeutic care option that offers advantages for people with AS when compared to UC at the primary care level. Despite promising outcomes favoring telerehabilitation over conventional treatment for AS, these results should be interpreted cautiously due to the scarcity of high-quality studies in this specific area. Future trials with robust methodologies are essential to validate our conclusions.

## Appendices

Ankle sprain rehabilitation exercise program: weekly progression (Protocol: Repeat exercise circuit to complete 30-minute daily sessions)

Exercises for Week 1: Focus on Range of Motion and Proprioception

1. Flex and extend the ankle vigorously and smoothly, repeating each movement 20 times.

2. Flex and extend toes, repeat each movement 20 times.

3. Wrinkle a towel with toes, repeat 5 times, and hold 2 seconds per contraction.

4. Plantar massage with ball: roll a ball with the plantar area, repeat it for 1 minute (gradual pressure).

5. Raise heels off of the ground and then go back down and repeat 20 times.

6. Stretch the calf muscles, leaning against a wall, do it for 30 seconds.

Exercises for Week 2: Focus on Range of Motion and Proprioception

1. Rotate the ankle and foot in circles to the right and to the left, 20 of each.

2. Perform eversion movement with the foot and ankle, 20 repetitions.

3. Perform inversion movement with the foot and ankle, 20 repetitions.

4. Make movements with the foot and ankle, forming the letters of the alphabet.

5. Walk on toes back and forward for 1 minute; if necessary, lean on a wall.

6. Walk on heels back and forward for 1 minute; if necessary, lean on a wall.

7. Standing on one leg, trying to maintain balance for 30 seconds to 1 minute.

8. On a pillow, stand on one leg, trying to maintain balance for 30 seconds to 1 minute.

Exercises for Weeks 3 and 4: Focus on Muscular Strengthening

1. Make a hoop with a bandage or elastic band, fix it on a piece of furniture, place the foot inside the hoop, and perform 30 dorsiflexion movements, applying resistance on the bandage or band.

2. Make a hoop with a bandage or elastic band, take it with the hand, place the foot inside the hoop, and perform 30 movements of plantar flexion, applying resistance on the bandage or band.

3. Make a hoop with a bandage or elastic band, take it with the hand, place the foot inside the hoop, and perform 30 movements of inversion, applying resistance on the bandage or band.

4. Make a hoop with a bandage or elastic band, take it with the hand, place the foot inside the hoop, and perform 30 movements of eversion, applying resistance on the bandage or band.

5. Make a hoop with a bandage or elastic band, place both feet inside the hoop, and perform 30 movements of eversion, applying resistance on the bandage or band.

## References

[1] Delahunt E, Bleakley CM, Bossard DS (2018). Clinical assessment of acute lateral ankle sprain injuries (ROAST): 2019 consensus statement and recommendations of the International Ankle Consortium. Br J Sports Med.

[2] Herzog MM, Kerr ZY, Marshall SW, Wikstrom EA (2019). Epidemiology of ankle sprains and chronic ankle instability. J Athl Train.

[3] Hiller CE, Nightingale EJ, Raymond J, Kilbreath SL, Burns J, Black DA, Refshauge KM (2012). Prevalence and impact of chronic musculoskeletal ankle disorders in the community. Arch Phys Med Rehabil.

[4] Vuurberg G, Hoorntje A, Wink LM (2018). Diagnosis, treatment and prevention of ankle sprains: Update of an evidence-based clinical guideline. Br J Sports Med.

[5] Chen ET, Borg-Stein J, McInnis KC (2019). Ankle sprains: Evaluation, rehabilitation, and prevention. Curr Sports Med Rep.

[6] van den Bekerom MP, Sjer A, Somford MP, Bulstra GH, Struijs PA, Kerkhoffs GM (2015). Non-steroidal anti-inflammatory drugs (NSAIDs) for treating acute ankle sprains in adults: Benefits outweigh adverse events. Knee Surg Sports Traumatol Arthrosc.

[7] Feger MA, Glaviano NR, Donovan L, Hart JM, Saliba SA, Park JS, Hertel J (2017). Current trends in the management of lateral ankle sprain in the United States. Clin J Sport Med.

[8] Turolla A, Rossettini G, Viceconti A, Palese A, Geri T (2020). Musculoskeletal physical therapy during the COVID-19 pandemic: Is telerehabilitation the answer?. Phys Ther.

[9] Correia FD, Molinos M, Neves C (2021). Digital rehabilitation for acute ankle sprains: Prospective longitudinal cohort study. JMIR Rehabil Assist Technol.

[10] (2023). Diagnóstico y manejo del esguince de tobillo en la fase aguda para el primer nivel de atención. http://www.imss.gob.mx/sites/all/statics/guiasclinicas/034GER.pdf.

[11] Martin RL, Irrgang JJ, Burdett RG, Conti SF, Van Swearingen JM (2005). Evidence of validity for the Foot and Ankle Ability Measure (FAAM). Foot Ankle Int.

[12] Hoch MC, Andreatta RD, Mullineaux DR, English RA, Medina McKeon JM, Mattacola CG, McKeon PO (2012). Two-week joint mobilization intervention improves self-reported function, range of motion, and dynamic balance in those with chronic ankle instability. J Orthop Res.

[13] van Rijn RM, van Ochten J, Luijsterburg PA, van Middelkoop M, Koes BW, Bierma-Zeinstra SM (2010). Effectiveness of additional supervised exercises compared with conventional treatment alone in patients with acute lateral ankle sprains: Systematic review. BMJ.

[14] Bleakley CM, O'Connor SR, Tully MA (2010). Effect of accelerated rehabilitation on function after ankle sprain: Randomised controlled trial. BMJ.

[15] Ortega-Avila AB, Cervera-Garvi P, Marchena-Rodriguez A, Chicharro-Luna E, Nester CJ, Starbuck C, Gijon-Nogueron G (2020). Conservative treatment for acute ankle sprain: A systematic review. J Clin Med.

[16] Hultman K, Fältström A, Öberg U (2010). The effect of early physiotherapy after an acute ankle sprain. Adv Physiother.

[17] Vargas FC, Ulate SG, Arce DP (2020). Conservative management of ankle sprains. (Article in Spanish). Rev Méd Sinerg.

[18] Gribble PA, Bleakley CM, Caulfield BM (2016). 2016 consensus statement of the International Ankle Consortium: Prevalence, impact and long-term consequences of lateral ankle sprains. Br J Sports Med.

[19] Medina McKeon JM, Bush HM, Reed A, Whittington A, Uhl TL, McKeon PO (2014). Return-to-play probabilities following new versus recurrent ankle sprains in high school athletes. J Sci Med Sport.

[20] Jones MH, Amendola AS (2007). Acute treatment of inversion ankle sprains: Immobilization versus functional treatment. Clin Orthop Relat Res.

[21] Polzer H, Kanz KG, Prall WC, Haasters F, Ockert B, Mutschler W, Grote S (2012). Diagnosis and treatment of acute ankle injuries: Development of an evidence-based algorithm. Orthop Rev.

[22] Vicente-Herrero M, Delgado-Bueno S, Bandrés-Moyá F, Capdevilla-García L: Valoración del dolor (2018). Pain assessment: A comparative review of scales and questionnaires. (Article in Spanish). J Spanish Pain Soc.

[23] van Rijn RM, van Os AG, Bernsen RM, Luijsterburg PA, Koes BW, Bierma-Zeinstra SM (2008). What is the clinical course of acute ankle sprains? A systematic literature review. Am J Med.

[24] Kelly AM (1998). Does the clinically significant difference in visual analog scale pain scores vary with gender, age, or cause of pain?. Acad Emerg Med.

[25] Todd KH, Funk KG, Funk JP, Bonacci R (1996). Clinical significance of reported changes in pain severity. Ann Emerg Med.

[26] Dupont M, Béliveau P, Thériault G (1987). The efficacy of antiinflammatory medication in the treatment of the acutely sprained ankle. Am J Sports Med.

[27] Solomon DH (2023). UpToDate: Nonselective NSAIDs: Overview of adverse effects. UpToDate.

[28] Bleakley CM, Taylor JB, Dischiavi SL, Doherty C, Delahunt E (2019). Rehabilitation exercises reduce reinjury post ankle sprain, but the content and parameters of an optimal exercise program have yet to be established: A systematic review and meta-analysis. Arch Phys Med Rehabil.

[29] Schulz KF, Altman DG, Moher D (2010). CONSORT 2010 Statement: Updated guidelines for reporting parallel group randomised trials. Trials.

